# Association of age-stratified cytokine profiles with clinical outcomes in patients with severe fever with thrombocytopenia syndrome

**DOI:** 10.1186/s12879-026-13105-z

**Published:** 2026-03-24

**Authors:** Chenxi Zhao, Ziruo Ge, Wenjing Niu, Ling Lin, Zishuai Liu, Tingyu Zhang, Jianping Duan, Hongxiao Wu, Ruihua Zhang, Yanli Xu, Wei Zhang, Peng Chen, Zhihai Chen

**Affiliations:** 1 National Key Laboratory of Intelligent Tracking and Forecasting for Infectious Diseases, Beijing Ditan Hospital, Capital Medical University, No. 8 Jingshun East Street, Chaoyang District, 100015 China; 2 Department of Infectious Diseases, Yantai Qishan Hospital, Yantai, Shandong Province 264001 China; 3 Department of Gastroenterology, Beijing Hospital, National Center of Gerontology, Institute of Geriatric Medicine, Chinese Academy of Medical Sciences, Beijing, 100370 China; 4 Department of Infectious Diseases, Qingdao Sixth People’s Hospital, No. 9, Fushun Road, Shibei District, Qingdao, Shandong Province 266033 China

**Keywords:** Severe fever with thrombocytopenia syndrome, Cytokine, Age

## Abstract

**Background:**

Severe fever with thrombocytopenia syndrome (SFTS) is a life-threatening zoonotic disease characterized by high morbidity and mortality. The aim of this study was to investigate the associations between serum cytokine levels and clinical outcomes in SFTS patients across different age groups to obtain deeper insights into disease pathogenesis.

**Methods:**

A total of 807 SFTS patients were enrolled in this two-center cohort study from January 2021 to December 2023. Serum samples from 78 of these patients were analyzed to determine the levels of 48 cytokines via the Luminex 200 platform. Patients were stratified by age and clinical outcome, and cytokine levels were compared across groups.

**Results:**

This study included 807 SFTS patients with an average age of 63.65 ± 10.63 years. Receiver operating characteristic (ROC) curve analysis was performed in the training cohort to evaluate the ability of age to predict clinical outcomes, and 66.5 years was identified as the optimal cutoff value (AUC 0.780, 95% CI 0.741–0.818). Based on this cutoff value and clinical outcomes, the 78 patients were stratified into four groups: older survivors, older non-survivors, younger survivors, and younger non-survivors. In this limited cohort, no significant differences in cytokine levels were observed between older and younger non-survivors, nor between older and younger survivors (FDR-adjusted q > 0.05). However, multiple cytokines were significantly elevated in non-survivors compared with survivors across age strata (q < 0.05), including representative cytokines such as IL-1β, IL-6, IL-10, IFN-γ, MCP-1, and MCP-3.

**Conclusion:**

Cytokine levels were associated with clinical outcomes in patients with SFTS. These findings suggest that cytokine profiles may serve as potential biomarkers reflecting disease severity. Early identification of high-risk patients and the exploration of cytokine-targeted therapies warrant further investigation.

**Clinical trial number:**

Not applicable.

**Graphical Abstract:**

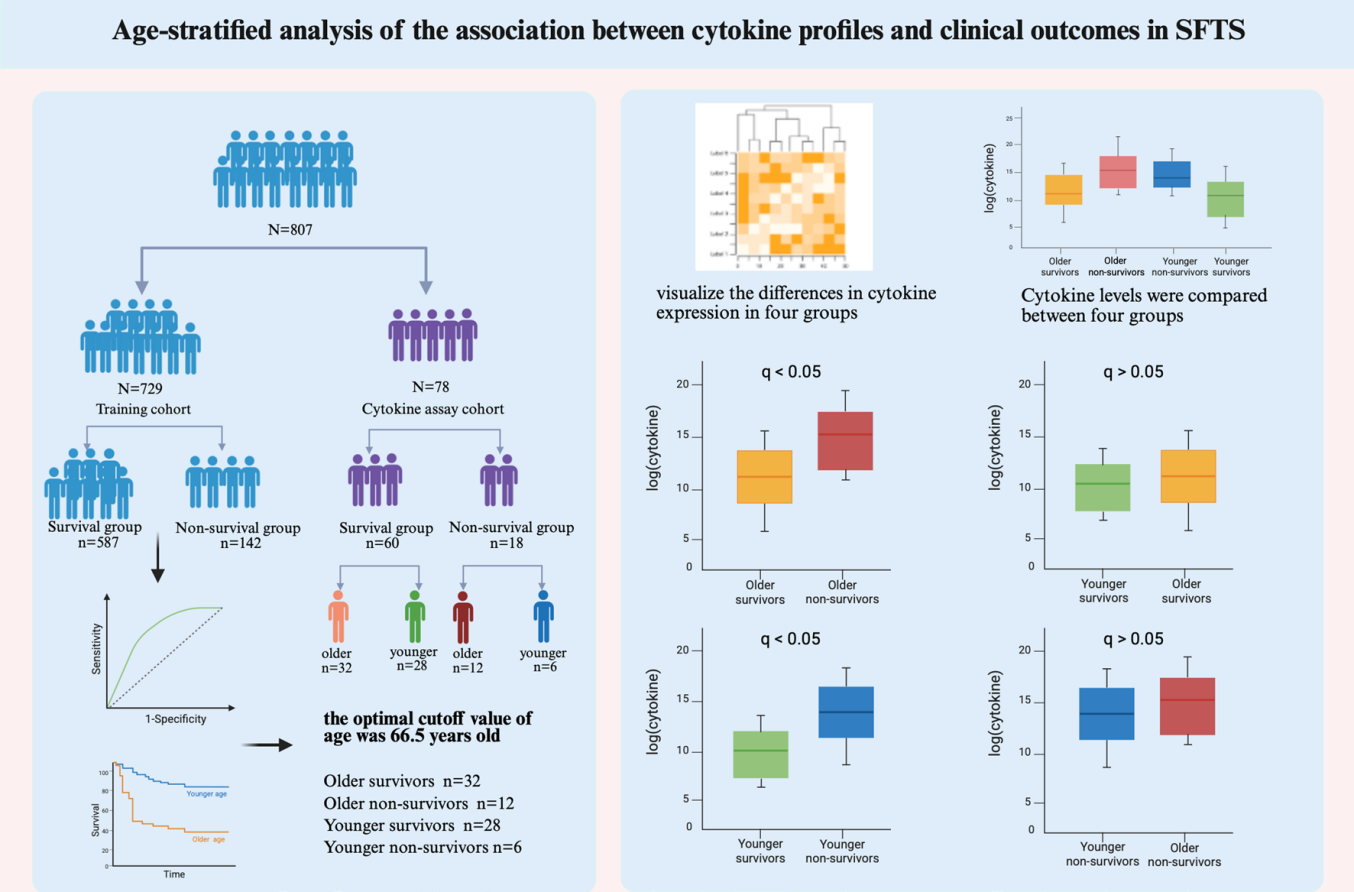

**Supplementary Information:**

The online version contains supplementary material available at 10.1186/s12879-026-13105-z.

## Background

Severe fever with thrombocytopenia syndrome (SFTS) is an emerging zoonotic infectious disease first reported in China that is caused by the SFTS virus (SFTSV) [1]. *Haemaphysalis longicornis* has been identified as the primary tick vector [2], with parthenogenetic reproduction playing a significant role in the rapid spread of SFTSV [3]. The virus has since spread to several countries, including South Korea, Japan, Vietnam, Thailand and Pakistan [4–8]. Initially, SFTSV infection was associated with a remarkably high case fatality rate (CFR) of 30% [1]. Recently, with increased understanding of SFTS and the introduction of the antiviral drug favipiravir [9], the current CFR ranges between 6.1% and 21.8% [10, 11]. In recent years, direct transmission of SFTSV from animals to humans has been reported, with this transmission involving cats in Japan, dogs in South Korea, and domestic sick camels in China [12–14]. More research is needed to address the high rates of infection and morbidity and high CFRs in SFTS, as well as to identify the factors influencing poor outcomes and the extent of their impact.

Numerous studies have investigated the risk factors associated with fatal outcomes in SFTS patients. Age has been identified as a crucial prognostic factor that significantly influences morbidity and mortality in SFTSV infection [15]. Although individuals of all age groups are susceptible to SFTS infection [16], the CFR increases with increasing age in SFTS patients [17]. Advanced age has been established as an independent risk factor for severe clinical outcomes [18, 19].

The aging process is likely closely associated with increased susceptibility to SFTSV infection, potentially through dysregulation of host immune cells and uncontrolled inflammatory responses [20]. An animal study demonstrated that older ferrets presented increased viral loads and more severe clinical symptoms following SFTSV infection. The upregulation of inflammatory pathway signaling through, for example, dendritic cell maturation, leukocyte extravasation, and IL-6 signaling, promotes the recruitment of inflammatory cells to infection sites, thereby exacerbating tissue damage and increasing mortality [21]. Previous pathophysiological and clinical studies have suggested that SFTSV-induced cytokine storms, coagulopathy, and multiorgan dysfunction contribute to the high CFRs observed for SFTS [22, 23]. Abnormal cytokine levels have been associated with increased severity and mortality in SFTS [24, 25].

Although age is a known prognostic factor in SFTS, the role of age-specific cytokine profiles and their impact on clinical outcomes has not been sufficiently explored. Age-related changes in immune function may result in differences in cytokine profiles, which in turn could influence disease severity. The aim of this study was to investigate serum cytokine levels in SFTS patients with different clinical outcomes across various age groups, with the goal of understanding the pathogenesis of SFTS and the underlying mechanisms involved.

## Methods

### Study design and patients

This two-center cohort study was conducted at Yantai Qishan Hospital and Qingdao Sixth People’s Hospital between January 2021 and December 2023. A total of 807 patients with confirmed SFTS were enrolled. The cytokine profiling was performed as a prospective sub-study at Yantai Qishan Hospital. From May to November 2022, consecutively admitted patients were included for cytokine analysis. The inclusion criteria for this study were as follows: (1) had a relevant epidemiological history; (2) had a positive real-time fluorescent polymerase chain reaction (RT‒PCR) test result for SFTSV RNA; and (3) were over 18 years of age. The exclusion criteria were (1) the presence of other viral infections (such as severe acute respiratory syndrome coronavirus 2 (SARS-CoV-2) and influenza virus infections); (2) positive test results for other tick-borne pathogens (including Hantaan virus*, Orientia tsutsugamushi,* or *Borrelia burgdorferi*); (3) a history of leukemia, idiopathic thrombocytopenic purpura, or other hematological and autoimmune diseases; (4) current treatment for malignant tumors due to the potential of these tumors to confound cytokine levels and clinical outcomes in SFTS patients; and (5) more than 10% of clinical data being missing. Patients were categorized into either the survivor group or the non-survivor group based on their clinical outcomes.

This study was approved by the Ethics Committee of Beijing Ditan Hospital, Capital Medical University (NO. DTEC-KY2022-022), and was conducted in accordance with the principles of the Declaration of Helsinki. All participants provided written informed consent.

### Data and sample collection

The patients’ baseline demographic information, clinical characteristics, and laboratory parameters were extracted from electronic medical records upon admission. For clinical variables with less than 10% missing data, missing values were imputed using the mean for normally distributed continuous variables, the median for non-normally distributed continuous variables, and the mode for categorical variables.

Peripheral blood samples were collected from 78 patients within 12 hours of admission during the acute phase of SFTS. Blood samples treated with ethylenediaminetetraacetic acid (EDTA) were centrifuged at 3000 rpm for 10 minutes, and the resulting serum was stored at −80 °C until further analysis. In this study, the acute phase was defined as the period within 7 days of symptom onset [26]. The observation endpoints were defined as survivor or death. Patients who were automatically discharged were followed up by phone calls one month after discharge to validate the clinical outcomes.

### Cytokine assay

The serum levels of 48 cytokines were quantified with the Bio-Plex Pro Human Cytokine Screening Panel 48-Plex (Bio-Rad, Hercules, California, USA; 12,007,283) according to the manufacturer’s instructions. Plate analysis was performed using the Luminex 200 platform (Milliplex Analyst, version 5.1). The original fluorescence units of each serum sample were applied to the standard curve to calculate the concentration of the sample in units of pg/mL. The following 48 cytokines were assayed: IL-1α, IL-1β, IL-1Ra, IL-2, IL- 2 Rα, IL-3, IL-4, IL-5, IL-6, IL-7, IL-9, IL-10, IL-12(p40), IL-12 (p70), IL-13, IL-15, IL-16, IL-17, IL-18, LIF, G-CSF, GM-CSF, M-CSF, SCF, SCGF-β, IFN-γ, IFN-α2, TNF-α, TNF-β, TRAIL, basic FGF, VEGF, PDGF-BB, HGF, β-NGF, CXCL1/GRO-α, CXCL8/IL-8, CXCL9/MIG, CXCL10/IP-10, CXCL12/SDF-1α, CCL2/MCP-1, CCL3/MIP-1α, CCL4/MIP-1β, CCL5/RANTES, CCL7/MCP-3, CCL11/eotaxin, CCL27/CTACK and MIF.

In the statistical analyses, cytokines were excluded if more than 50% of the patients had values below or above the detection limit [27]. For concentrations below the lowest detection limit, 50% of the lowest detection value was used [28].

### Statistical analysis

Data with a normal distribution are presented as the mean ± standard deviation and were compared between groups using the Student’s t test. Data that were not normally distributed are presented as median (interquartile range, IQR) and were compared using the Mann–Whitney U test. Categorical variables are presented as percentages and were compared using the χ^2^ test or Fisher’s exact test. The discriminative performance of age was evaluated using receiver operating characteristic (ROC) curve analysis. Hierarchical clustering analysis was performed to explore patterns in cytokine expression, and the results were visualized using heatmaps. To account for multiple comparisons in cytokine analyses, p values were adjusted using the Benjamini–Hochberg false discovery rate (FDR) method. All tests were two-sided, with *p* < 0.05 considered statistically significant and FDR-adjusted q < 0.05 applied for cytokine analyses. Statistical analyses were performed using SPSS software (version 29.0.1.0; IBM Corp., Armonk, NY, USA), and figures were generated using the R programming language (version 4.3.1).

## Results

### Baseline demographic characteristics of the patients

A total of 807 patients with SFTS were included in this study. The mean age was 63.65 ± 10.63 years, and 426 (52.8%) were male. The overall CFR was 19.8% (160/807). The study population was divided into a training cohort (*n* = 729) and a cytokine assay cohort (*n* = 78). No significant differences were observed between the two cohorts in terms of sex, age, or CFR (Supplementary Table S1).

Within the cytokine assay cohort, age, sex, time from onset to admission, and peak temperature were comparable between survivors and non-survivors (Table 1). Non-survivors exhibited significantly higher aspartate aminotransferase (AST) levels (*p* < 0.001), alanine aminotransferase (ALT) levels (*p* = 0.005), lactate dehydrogenase (LDH) levels (*p* < 0.001), activated partial thromboplastin time (APTT) (*p* = 0.002), D-dimer levels (*p* = 0.001), and viral load (*p* = 0.005). White blood cell (WBC) counts, platelet (PLT) counts and albumin levels were reduced in survivors and non-survivors, with no statistically significant differences between the two groups (*p* = 0.334, *p* = 0.066, and *p* = 0.295, respectively).

**Table 1:** Baseline demographic characteristics of the cytokine assay cohort

	Patients (*n* = 78)	Survivor group (*n* = 60)	Non-survivor group (*n* = 18)	*P* value
Sex, male, n (%)	37(47.4)	27(45.0)	10(55.6)	0.432
Age (years)	65.89 ± 11.19	64.92 ± 11.76	69.11 ± 8.54	0.165
Time from onset to admission (d)	5.88 ± 1.73	5.88 ± 1.81	5.89 ± 1.49	0.990
Highest temperature (°C)	38.71 ± 0.67	38.73 ± 0.71	38.63 ± 0.53	0.570
White blood cell(10^9^/L)	2.90(1.76–4.18)	2.81(1.62–4.19)	3.06(2.25–4.41)	0.334
Platelet(10^9^/L)	59.00(46.75–78.25)	61.00(49.00–82.00)	51.00(42.00–64.75)	0.066
Hemoglobin(g/L)	140.05 ± 18.24	138.30 ± 17.44	145.89 ± 20.12	0.122
AST(U/L)	151.50(74.20–331.28)	120.25(61.55–205.28)	379.50(170.00–722.53)	<0.001
ALT(U/L)	65.00(38.78–134.63)	52.70(38.30–111.78)	133.25(64.90–248.35)	0.005
Albumin(g/L)	30.92 ± 4.90	31.24 ± 4.85	29.86 ± 5.06	0.295
LDH(U/L)	611.00(370.00–980.25)	522.00(353.25–819.00)	1042.50(714.73–1829.75)	<0.001
APTT(s)	49.85(43.90–58.65)	47.15(43.38–55.60)	58.70(51.23–66.00)	0.002
D-dimer(μg/ml)	2.67(1.63–4.57)	2.45(1.38–3.42)	5.70(2.49–8.52)	0.001
Viral load(TCID_50_/ml)	1899.00(420.25–3780.50)	925.50(404.75–2633.75)	3858.00(2048.75–4384.00)	0.005

AST, aspartate aminotransferase; ALT, alanine aminotransferase; LDH, lactate dehydrogenase; APTT, activated partial thromboplastin time

### Analysis of the ROC curve revealed the best cutoff value for age

**Fig. 1:**
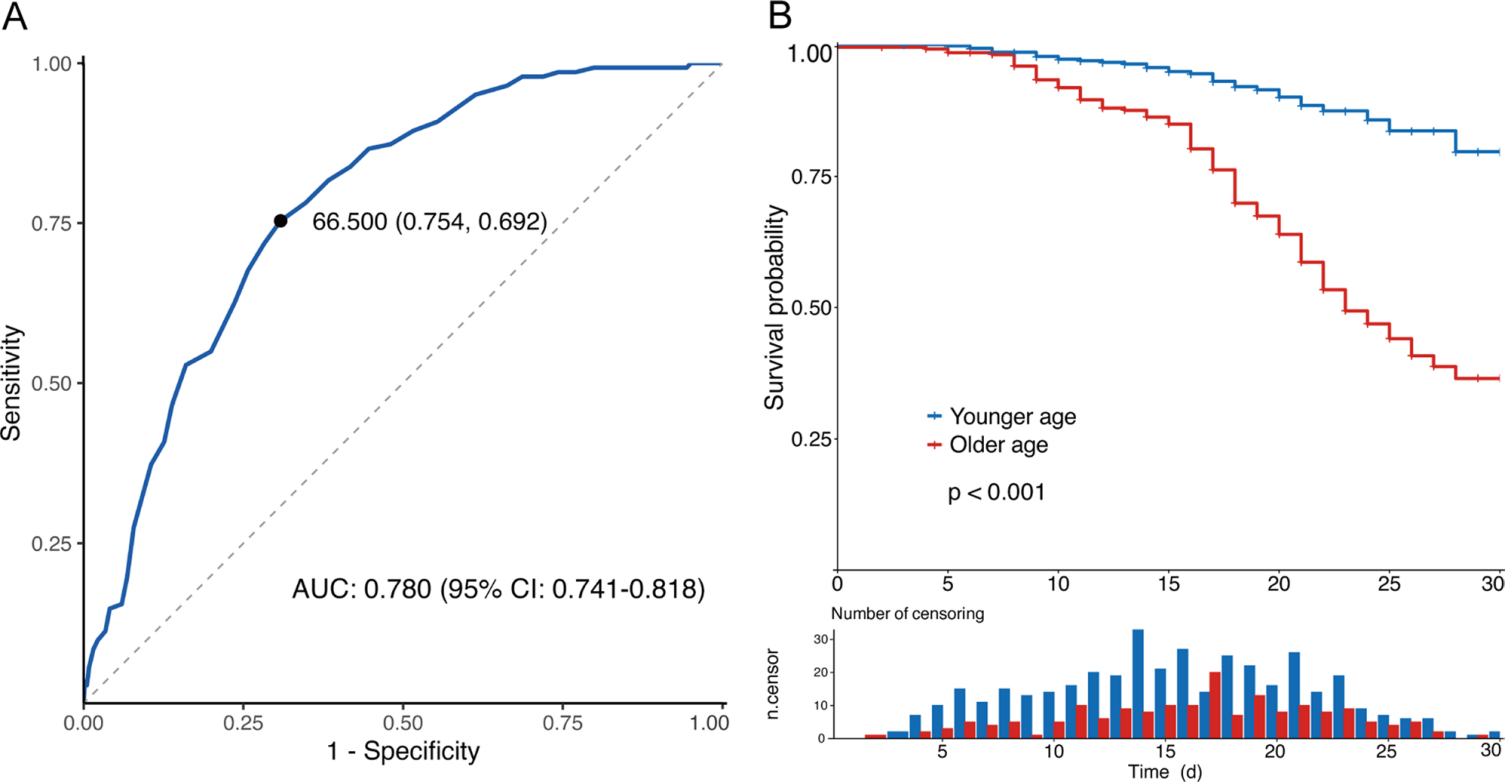
ROC curve and KM survival analysis of age in SFTS patients. (**A**) ROC curve analysis was performed in the training cohort to evaluate the ability of age to predict clinical outcomes. The optimal cutoff value was 66.5 years, with a sensitivity of 0.754 and a specificity of 0.692. (**B**) KM survival analysis based on the age cutoff value. *p* values were calculated via log-rank tests. ROC, receiver operating characteristic; KM, Kaplan–Meier; SFTS, severe fever with thrombocytopenia syndrome; AUC, area under the ROC curve

In the training cohort, ROC curve analysis was performed to evaluate the ability of age to predict clinical outcomes, yielding an area under the ROC curve (AUC) of 0.780, 95% confidence interval (CI): 0.741–0.818. According to the maximum Youden index, the optimal cutoff value was 66.5 years, with a sensitivity of 0.754 and a specificity of 0.692. This cutoff was subsequently used to stratify patients into older and younger groups (Fig. 1A).

Based on the optimal cutoff value for age, 729 patients were stratified into an older age group (≥67 years) and a younger age group (≤66 years). We further adopted the Kaplan‒Meier (KM) analysis method to estimate cumulative survival. The results indicated that patients with SFTS in the older age group had significantly worse survival than those in the younger age group (*p* < 0.001) (Fig. 1B).

### Heatmap analysis of cytokine profiles in SFTSV-infected patients

Serum samples from 78 patients with SFTS in the acute phase were assayed to determine the levels of 48 cytokines. Among these, 8 cytokines, including IL-2, IL-3, IL-5, IL-7, IL-12 (p70), IL-15, VEGF, and β-NGF, were excluded from the analysis because more than 50% of their levels were below the lower detection limit. A total of 78 patients with SFTS were categorized into 4 groups based on the optimal age cutoff value and clinical outcome: the older survivor group (*n* = 32), older non-survivor group (*n* = 12), younger survivor group (*n* = 28), and younger non-survivor group (*n* = 6) (Table 2).

**Table 2:** Comparison of age and sex among the four groups

	Older (>66 years)			Younger (≤66 years)		
	Survivor (*n* = 32)	Non-survivor (*n* = 12)	*P*^a^	Survivor (*n* = 28)	Non-survivor (*n* = 6)	*P*^b^
Sex, male, n (%)	12 (37.5)	7 (58.3)	0.214	15 (53.6)	3 (50.0)	1.000
Age (years)	73.88 ± 6.25	74.25 ± 3.66	0.490	54.68 ± 7.32	58.83 ± 5.35	0.257

*P*^a^: Comparison between the survivor and non-survivor groups in the older population

*P*^b^: Comparison between the survivor and non-survivor groups in the younger population

**Fig. 2:**
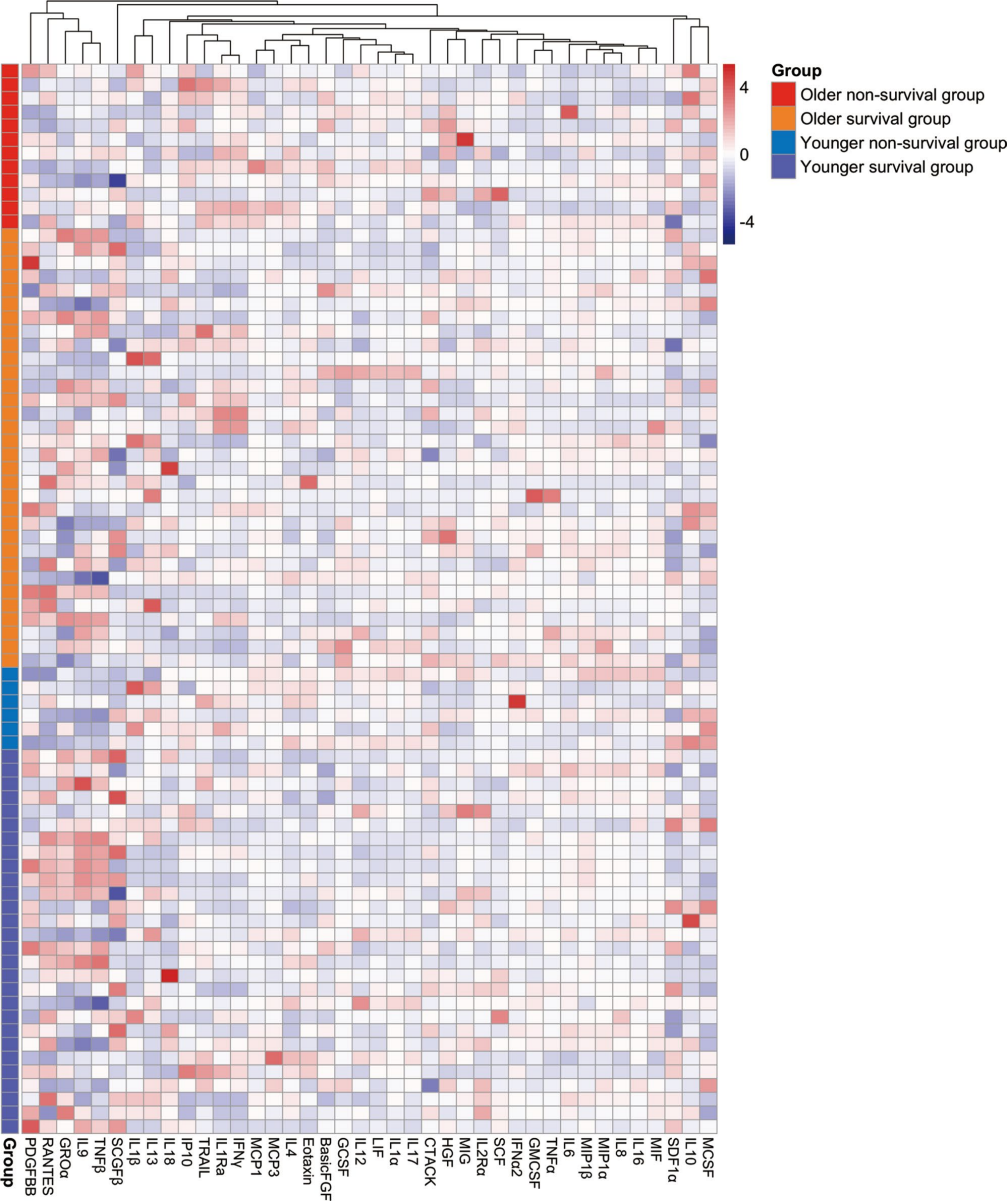
Heatmap analysis of cytokine profiles in patients with SFTS. The heatmap illustrates the expression levels of 40 cytokines measured in serum samples from 78 patients with SFTS. Navy to red colors in the heatmap represent low to high expression levels, respectively. White indicates median expression levels. Hierarchical clustering was performed using Euclidean distance and complete linkage

Serum cytokine levels were compared between survivors and non-survivors, and the results for all 40 cytokines are provided in Supplementary Table S2. To visualize the differences in cytokine expression levels, hierarchical clustering analysis was performed using a heatmap, which was then used to classify the 40 cytokines across the four patient groups (Fig. 2).

### Comparison of cytokine levels between older and younger non-survivors

**Fig. 3:**
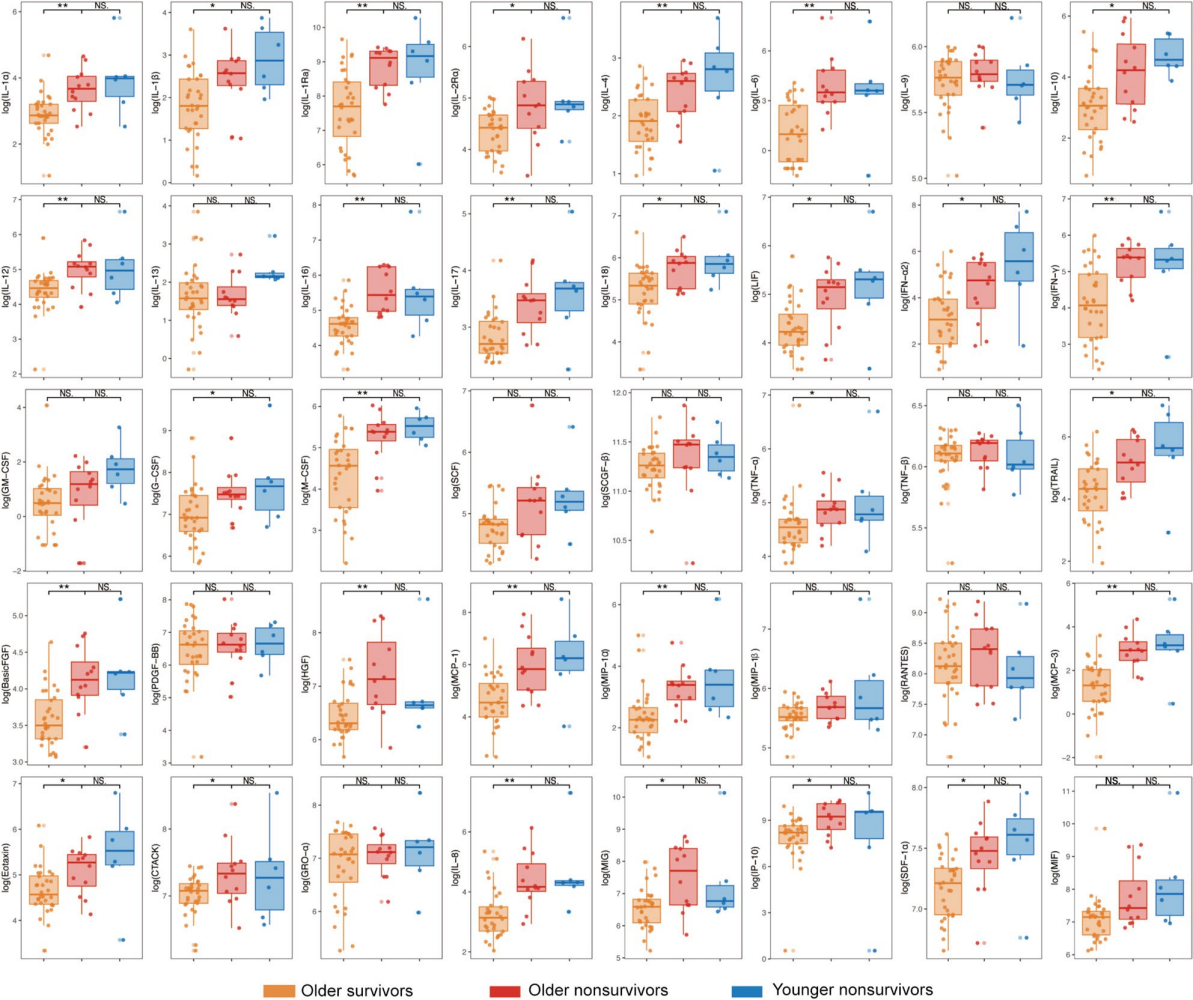
Distribution of cytokine levels among older survivors, older non-survivors, and younger non-survivors. Cytokine levels were compared between older and younger non-survivors and between older survivors and non-survivors. The cytokine data were logarithmically transformed for analysis and comparison. In the box plots, the central line represents the median, and the upper and lower edges indicate the 75th and 25th percentiles (IQR), respectively. *p* values were adjusted for multiple comparisons using the benjamini–Hochberg false discovery rate (FDR) method, and statistical significance is indicated based on FDR-adjusted q values. * q < 0.05, ** q < 0.01, *** q < 0.001. NS, no significant difference

When comparing older and younger non-survivors, none of the 40 cytokines reached statistical significance after FDR adjustment (q > 0.05) in this limited cohort (Fig. 3). However, this analysis was based on a relatively small younger non-survivor subgroup.

### Comparison of cytokine levels between older survivors and non-survivors

Among older patients, twenty-nine cytokines showed statistically significant differences between survivors and non-survivors after FDR adjustment (q < 0.05), with higher levels observed in non-survivors (Fig. 3). Representative cytokines included IL-1β, IL-6, IL-10, IL-17, IFN-γ, MCP-1, MCP-3, and IP-10.

### Comparison of cytokine levels between older and younger survivors

**Fig. 4:**
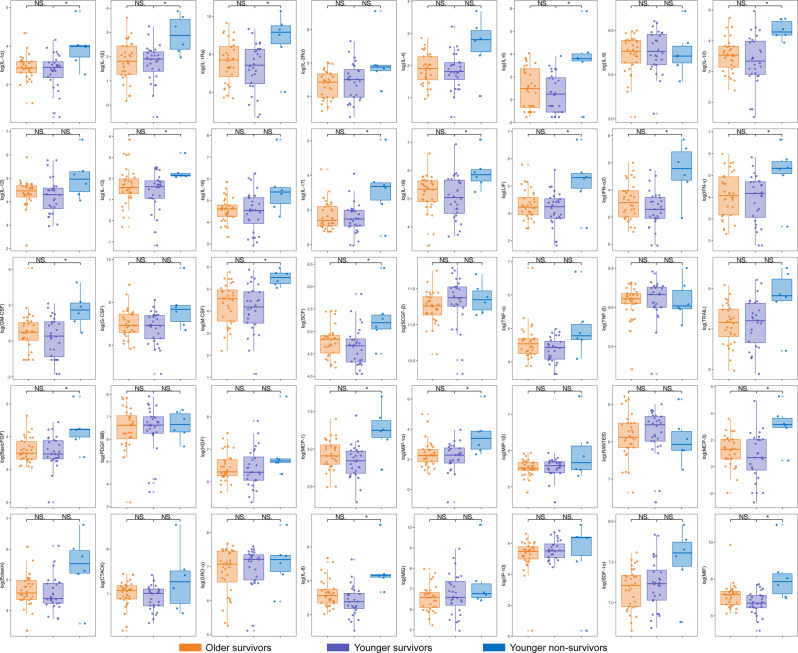
Distribution of cytokine levels among older survivors, younger survivors, and younger non-survivors. Cytokine levels were compared between older and younger survivors, as well as between younger survivors and non-survivors. The data were logarithmically transformed for analysis. In the box plots, the central line represents the median, whereas the upper and lower edges correspond to the 75th and 25th percentiles (IQR), respectively. *p* values were adjusted for multiple comparisons using the benjamini–Hochberg false discovery rate (FDR) method, and statistical significance is indicated based on FDR-adjusted q values. * q < 0.05, ** q < 0.01, *** q < 0.001. NS, no significant difference

When comparing older and younger survivors, none of the 40 cytokines reached statistical significance after FDR adjustment (q > 0.05) in this cohort (Fig. 4).

### Comparison of cytokine levels between younger survivors and non-survivors

Among younger patients, 20 cytokines showed statistically significant differences between survivors and non-survivors after FDR adjustment (q < 0.05), with higher levels observed in non-survivors (Fig. 4). Representative cytokines included IL-1β, IL-6, IL-10, IL-17, IFN-γ, GM-CSF, MCP-1, and MCP-3.

## Discussion

SFTS is a severe infectious zoonotic disease with high morbidity and mortality. Previous studies have identified advanced age as a key prognostic factor associated with adverse outcomes in patients with SFTS, with a markedly increased CFR [29, 30]. For example, Zhang et al. reported that the risk of death in SFTS patients over 65 years of age was 3.384 times greater than that in younger patients [31]. In our study, ROC curve analysis in the training cohort identified 66.5 years as the optimal age cutoff for predicting clinical outcomes, and this threshold was subsequently used for age stratification in the present study. It was not intended to replace the conventional clinical age threshold of 65 years, and its clinical applicability should therefore be interpreted cautiously and validated in independent cohorts. Consistent with previous studies, our findings support the association between advanced age and higher CFR in SFTS, possibly reflecting age-related immune dysfunction and other age-related host factors that may contribute to poor clinical outcomes [32].

Although previous studies have revealed higher complication rates and CFRs among male SFTS patients [29], our study did not reveal a significant difference in CFR between the sexes, which aligns with some earlier research findings [33]. The relationship between sex and disease severity, including the magnitude of cytokine storms, remains inconsistent across studies. Further investigation is warranted to determine whether sex-based differences truly exist in immune responses to SFTSV infection.

Previous studies have established cytokine storms as a hallmark pathological feature of severe SFTS [22], contributing to systemic inflammatory symptoms, coagulation abnormalities, and multiorgan dysfunction [34, 35]. SFTSV infection has been shown to trigger excessive immune activation during the acute phase, characterized by abnormal expression of multiple cytokines, which is closely associated with disease severity [24, 36]. Heatmap analysis and hierarchical clustering demonstrated distinct cytokine expression patterns across the study groups, suggesting an association between cytokine dysregulation and disease progression. In the acute phase, non-survivors exhibited broadly elevated inflammatory cytokine levels compared with survivors. Representative cytokines included IL-1β, IL-6, IL-10, IL-17, IFN-γ, MCP-1, and MCP-3, which are involved in inflammatory and immune signaling pathways. These abnormally elevated cytokine levels in non-survivors are similar to those reported in previous studies [22, 37, 38]. The marked elevation of these cytokine levels in non-survivors highlights the critical role of cytokine dysregulation in the progression of SFTS. In a case report of SFTS in which the patient died, persistently elevated levels of TNF-α, IL-6, IP-10, and granzyme B were recorded, despite repeated plasma exchange treatments [39].

Although age was associated with mortality in our cohort, age-related differences in cytokine levels were not statistically significant in the subgroup analyses. Specifically, no significant differences were observed between older and younger non-survivors or between older and younger survivors after FDR adjustment. Elevated cytokine levels were observed in non-survivors across age strata in this cohort. Given the limited subgroup sizes, particularly among younger non-survivors, these findings should be interpreted with caution. The mechanisms underlying the poorer outcomes observed in older patients remain unclear. Previous studies have suggested that age-related physiological changes and comorbid conditions may influence disease severity in SFTS [40]. Monocytes from older individuals have been reported to be more susceptible to SFTSV infection and phenotypic alterations [30], and such age-related immune changes may increase vulnerability in elderly individuals [41]. These factors were not directly assessed in the present study.

Given the important role of cytokine dysregulation in SFTS pathogenesis, cytokine profiling holds promise for both prognosis and targeted therapy. A study revealed that patients with SFTS-associated encephalopathy or encephalitis tested positive for SFTSV RNA and presented elevated levels of MCP-1and IL-8 in their cerebrospinal fluid [42]. Early cytokine profiling may enable the identification of patients at high risk for progression to severe disease or fatal outcomes, regardless of age. Elucidating the specific roles of cytokines in SFTS pathogenesis could guide the development of targeted therapeutic strategies aimed at modulating the immune response and mitigating the effects of cytokine storms. Ruxolitinib, a Janus kinase (JAK) 1/2 inhibitor that blocks proinflammatory cytokines and suppresses the type I interferon signaling pathway, has shown promise in reducing the incidence of severe SFTS and decreasing intensive care unit (ICU) admission rates [43]. Targeting specific cytokines or inflammatory pathways may offer a promising therapeutic strategy. Among these, IL-6 is a pleiotropic cytokine with proinflammatory effects in vivo that activates various signaling pathways, including the JAK/STAT3, Ras/MAPK, and PI3K-Akt pathways. IL-6 plays a crucial role in the pathogenesis of various diseases and conditions, including cancer, infectious diseases, and autoimmune disorders [44]. A case study revealed that IL-6 levels in an SFTS patient peaked on the day of admission and subsequently declined dramatically following the administration of tocilizumab, an anti-IL-6 receptor monoclonal antibody. This intervention was associated with notable clinical improvement and normalization of laboratory parameters, without any serious adverse events [45]. These findings highlight the potential of cytokine-targeted therapies and suggest that cytokine profiling may contribute not only to individualized treatment planning but also to real-time monitoring of therapeutic efficacy.

Our findings provide further insight into the immunopathological characteristics associated with severe outcomes in patients with SFTS. Age remained an important prognostic factor in this cohort. In the cytokine sub-study, elevated cytokine levels were observed in non-survivors across age groups. No statistically significant differences were identified between older and younger non-survivors in this relatively small cohort. Overall, the results support an association between cytokine dysregulation and adverse clinical outcomes in SFTS. Cytokine profiling may reflect disease severity and may have potential value for risk stratification. Confirmation in larger, multicenter cohorts is required. From a clinical perspective, earlier recognition of patients at risk of excessive inflammatory responses may allow closer monitoring and more timely, individualized management.

## Limitations

Our study has several limitations. First, the cytokine analysis was conducted at a single center within a defined period, which may limit the generalizability of the findings. Second, the younger non-survivor subgroup was small (*n* = 6), limiting statistical power and increasing the risk of type II error in age-stratified comparisons; these findings should therefore be interpreted with caution. Third, not all cytokines were detectable in every sample, which may introduce potential bias. Finally, external validation was not conducted. Future studies with larger, multicenter cohorts and longitudinal cytokine tracking are needed to validate and extend these findings.

## Conclusion

Our results indicate that age is an important prognostic factor in patients with SFTS. In this cohort, no statistically significant differences in cytokine levels were observed between older and younger non-survivors. Several cytokines differed significantly between survivors and non-survivors across age strata. These findings support an association between cytokine dysregulation and adverse clinical outcomes. Cytokine profiling may help reflect disease severity, and its potential clinical value warrants further investigation in larger studies.

## Electronic supplementary material

Below is the link to the electronic supplementary material.

Supplementary Material 1

## Data Availability

The data supporting the findings of this study are available from the corresponding author upon reasonable request.

## Abbreviations

SFTS: Severe fever with thrombocytopenia syndrome
SFTSV: Severe fever with thrombocytopenia syndrome virus
CFR: Case fatality rate
RT‒PCR: Real-time fluorescent polymerase chain reaction
EDTA: Ethylenediaminetetraacetic acid
IQR: Interquartile range
ROC: Receiver operating characteristic
AUC: Area under the curve
IL: Interleukin
LIF: Leukemia inhibitory factor
G-CSF: Granulocyte colony stimulating factor
GM-CSF: Granulocyte–macrophage colony-stimulating factor
M-CSF: Macrophage colony stimulating factor
SCF: Stem cell factor
SCGF-β: Stem cell growth factor beta
IFN: Interferon
TNF: Tumor necrosis factor alpha
TRAIL: TNF-related apoptosis-inducing ligand
Basic FGF: Basic fibroblast growth factor
VEGF: Vascular endothelial growth factor
PDGF-BB: Platelet-derived growth factor-BB
HGF: Hepatocyte growth factor
CXCL: C-X-C motif chemokine ligand
GRO-α: Growth-related oncogene alpha
MIG: Monokine induced by gamma interferon
IP-10: Interferon inducible protein 10
SDF-1α: Stromal cell-derived factor 1alpha
MCP: Monocyte chemoattractant protein
MIP: Macrophage inflammatory protein
RANTES: Regulated upon activation, normal T cells are expressed and presumably secreted
CTACK: Cutaneous T-cell attracting chemokine
MIF: Macrophage migration inhibitory factor
KM: Kaplan‒Meier
