# Supplementary material for: Association of age-stratified cytokine profiles with clinical outcomes in patients with severe fever with thrombocytopenia syndrome
Source: BMC Infect Dis. 2026 Mar 24;26:868. doi: 10.1186/s12879-026-13105-z (PMC13134252; doi:10.1186/s12879-026-13105-z)
Supplement: Supplementary file 1 — Supplementary Material 1 [file 12879_2026_13105_MOESM1_ESM.docx]

Supplementary

Table S1. Baseline demographic characteristics of the 807 patients with SFTS

|  | Total  (n = 807) | Training Cohort  (n = 729) | | Cytokine Assay Cohort  (n = 78) | | *P* value |
| --- | --- | --- | --- | --- | --- | --- |
|  |  | Survivor group  (n = 587) | Non-survivor group  (n = 142) | Survivor group  (n = 60) | Non-survivor group  (n = 18) |  |
| Sex, male, n (%) | 426(52.8) | 305 (52.0) | 84 (59.2) | 27(45.0) | 10(55.6) | 0.319 |
| Age, years | 63.65$\pm$10.63 | 61.46$\pm$10.17 | 71.50$\pm$7.94 | 64.92$\pm$11.76 | 69.11$\pm$8.54 | 0.051 |
| <40 | 9 (1.1) | 9 (1.5) | 0 (0) | 0 (0) | 0 (0) | 1.000 |
| 40-49 | 76 (9.4) | 67 (11.4) | 1 (0.7) | 7 (11.7) | 1 (5.6) | 0.790 |
| 50-59 | 211 (26.1) | 186 (31.7) | 12 (8.5) | 12 (20.0) | 1 (5.6) | 0.045 |
| 60-69 | 252 (31.2) | 186 (31.7) | 40 (28.2) | 19 (31.7) | 7 (38.9) | 0.673 |
| 70-79 | 208 (25.8) | 115 (19.6) | 68 (47.9) | 16 (26.7) | 9 (50.0) | 0.182 |
| ≥80 | 51 (6.3) | 24 (4.1) | 21 (14.8) | 6 (10.0) | 0 (0) | 0.600 |
| Mortality | 19.8% | 19.5% | | 23.1% | | 0.449 |

*P*: Comparison of sex, age and mortality rate between the training cohort and the cytokine assay cohort.

Table S2. Comparison of serum cytokine levels between survivors and non-survivors

|  | Survivor group  (n = 60) | Non-survivor group  (n = 18) | *P* value | q value (FDR) |
| --- | --- | --- | --- | --- |
| IL-1Ra | 1959.50 (652.58, 3952.00) | 9351.00 (4229.00, 11052.25) | <0.001 | <0.001 |
| IL-6 | 1.98 (0.34, 11.70) | 36.38 (20.63, 107.47) | <0.001 | <0.001 |
| IL-16 | 96.38 (62.12, 142.89) | 223.26 (138.25, 488.19) | <0.001 | <0.001 |
| IL-17 | 15.46 (12.67, 20.36) | 32.80 (23.37, 42.09) | <0.001 | <0.001 |
| IFN-γ | 63.71 (23.50, 112.67) | 216.37 (134.65, 298.00) | <0.001 | <0.001 |
| M-CSF | 86.60 (33.92, 137.57) | 217.99 (186.05, 290.18) | <0.001 | <0.001 |
| Basic FGF | 32.30 (27.55, 48.12) | 67.06 (50.40, 72.13) | <0.001 | <0.001 |
| MCP-1 | 87.56 (43.17, 159.64) | 424.32 (187.06, 1028.13) | <0.001 | <0.001 |
| MIP-1α | 9.62 (5.90, 15.01) | 29.71 (16.27, 44.53) | <0.001 | <0.001 |
| MCP-3 | 2.75 (1.18, 7.71) | 19.14 (12.55, 30.16) | <0.001 | <0.001 |
| IL-8 | 19.10 (12.32, 30.95) | 70.16 (60.12, 113.36) | <0.001 | <0.001 |
| IL-1α | 17.55 (12.40, 24.63) | 43.63 (27.04, 57.18) | <0.001 | <0.001 |
| IL-4 | 6.50 (4.75, 8.60) | 14.14 (8.20, 16.87) | <0.001 | <0.001 |
| IL-10 | 19.03 (7.41, 41.20) | 79.13 (37.61, 204.06) | <0.001 | <0.001 |
| IL-18 | 193.19 (110.34, 280.99) | 357.11 (213.10, 423.42) | <0.001 | <0.001 |
| IFN-α2 | 16.43 (6.86, 38.56) | 147.30 (46.04, 296.68) | <0.001 | <0.001 |
| IL-1β | 6.38 (3.57, 9.38) | 13.12 (9.54, 18.48) | <0.001 | <0.001 |
| LIF | 68.50 (48.38, 95.28) | 186.89 (122.51, 227.82) | <0.001 | <0.001 |
| MIF | 1017.83 (649.60, 1440.75) | 1956.00 (1166.50, 4001.25) | <0.001 | <0.001 |
| IL-12 | 79.04 (49.95, 105.52) | 160.79 (95.65, 196.73) | <0.001 | <0.001 |
| HGF | 541.72 (461.82, 824.61) | 864.86 (729.15, 2050.25) | <0.001 | 0.001 |
| Eotaxin | 91.21 (76.44, 135.72) | 203.79 (128.74, 246.22) | <0.001 | 0.002 |
| G-CSF | 1012.50 (651.44, 1656.25) | 1840.50 (1380.00, 2554.50) | 0.001 | 0.002 |
| TNF-α | 85.70 (66.42, 108.53) | 130.08 (103.01, 164.66) | 0.001 | 0.002 |
| TRAIL | 78.07 (29.18, 156.10) | 217.99 (115.86, 396.87) | 0.001 | 0.002 |
| SDF-1α | 1367.00 (1074.00, 1595.00) | 1872.50 (1619.00, 2091.25) | 0.001 | 0.002 |
| SCF | 112.22 (79.69, 132.99) | 183.76 (117.25, 222.12) | 0.002 | 0.003 |
| IL-2Rα | 85.64 (53.02, 111.95) | 128.72 (90.70, 191.04) | 0.003 | 0.005 |
| GM-CSF | 1.58 (0.60, 2.93) | 3.73 (2.04, 6.62) | 0.004 | 0.005 |
| CTACK | 1131.50 (885.56, 1282.00) | 1531.50 (1081.50, 1821.00) | 0.007 | 0.010 |
| MIG | 726.24 (440.91, 1082.25) | 1246.22 (765.15, 4190.25) | 0.009 | 0.011 |
| IP-10 | 3710.00 (1940.75, 6850.25) | 12529.00 (3564.00, 22142.25) | 0.009 | 0.011 |
| MIP-1β | 255.19 (220.38, 295.69) | 294.48 (242.21, 365.45) | 0.057 | 0.069 |
| IL-13 | 4.91 (2.99, 6.94) | 6.81 (4.54, 9.39) | 0.090 | 0.106 |
| SCGF-β | 82122.67 ± 21448.98 | 89295.56 ± 26131.35 | 0.299 | 0.341 |
| IL-9 | 319.74 ± 65.40 | 330.78 ± 66.59 | 0.541 | 0.601 |
| GRO-α | 1261.00 (741.15, 1651.00) | 1230.50 (925.89, 1531.25) | 0.785 | 0.849 |
| PDGF-BB | 758.91 (505.18, 1106.00) | 754.22 (559.76, 1179.50) | 0.817 | 0.860 |
| RANTES | 3720.50 (2663.50, 5377.50) | 4206.00 (2391.00, 5823.75) | 0.901 | 0.924 |
| TNF-β | 458.41 (404.90, 513.63) | 462.52 (400.47, 503.31) | 0.934 | 0.934 |

Cytokine levels are presented as mean ± standard deviation for normally distributed variables and as median (interquartile range) for non-normally distributed variables. Between-group comparisons were performed using the t test or the Mann–Whitney U test. To account for multiple comparisons across 40 cytokines, P values were adjusted using the Benjamini–Hochberg false discovery rate (FDR) method. Two-sided P and q values are reported.
